# Defaulting peripherally inserted central catheter (PICC) orders to single lumen: A quality improvement initiative at a Midwestern academic center 2022–2023

**DOI:** 10.1017/ash.2023.511

**Published:** 2023-12-18

**Authors:** Kathryn Trautman, Takaaki Kobayashi, Chad McPherson, Karen Brust

**Affiliations:** Quality Improvement Program, University of Iowa Hospitals & Clinics, Iowa City, IA, USA

## Abstract

Defaulting the order for peripherally inserted central catheters (PICCs) placement to single lumen increased proportion of single-lumen insertions over total insertions from 42/126 (33%) to 57/104 (51)%. Single-lumen PICCs had a nonsignificant lower rate of central line-associated bloodstream infection compared to double-lumen PICCs.

## Introduction

Peripherally inserted central catheters (PICCs) have become the preferred choice for central access due to their expanded indications for patients requiring long-term access for vasopressors, central nutrition, chemotherapy, and vesicant medications.^1^ PICC placement is often considered easier, safer, and more durable compared to traditional central venous catheters (CVCs). However, despite these advantages, PICC-related central line-associated bloodstream infections (CLABSIs) present ongoing challenges.^2^ Several factors contribute to increased risk of CLABSI development, including longer duration of central line being in place, emergent or noncontrolled conditions of insertion, lower expertise of insertion operator, and missed opportunities of catheter-site care. Likewise, specific line-related characteristics will increase CLABSI risk such as nonantimicrobial coated catheter and increased number of catheter lumens.^2,3^ Each additional PICC lumen increases the risk of infection. However, these data are confounded by the fact that sicker patients tend to receive CVCs with more lumens, leading to increased access through each port.^4^ More recent data suggest a statistical higher risk of CLABSI between one and three PICC lumens, but the evidence is less certain between one and two PICC lumens.^5^

Our institutional analysis revealed that the majority of adult PICC lines ordered and placed were double-lumen catheters, even when a single lumen would have sufficed. This highlighted an opportunity to enhance assessment criteria and ordering practices, promoting the use of single-lumen PICCs instead of ordering double-lumen PICCs. The quality improvement program led an effort to develop an electronic medical record (EMR) “nudge” to encourage the ordering of single-lumen PICCs by default, with the aim of improving PICC-ordering practices within the institution.

## Methods

We conducted a quality improvement initiative at University of Iowa Hospitals and Clinics between March 1, 2022, and June 30, 2023. This EMR intervention was led by the CLABSI Core Team, with experts from the quality improvement program, vascular access, infection prevention, and the program of hospital epidemiology. The EMR change “nudged” ordering providers to select single-lumen PICCs by highlighting the single-lumen PICC option (Supplemental Figure 1). Hospital epidemiology met with the adult vascular access nursing team as the primary PICC inserters, and the order change was shared with both medical and nursing staff. Along with the EMR change, education was provided at multiple forums on the risk reduction related to lower number of central line lumens to further influence this practice change. Outcome measures included the proportion of single- versus double-lumen PICC placements and line-days. These included PICC placements by the adult vascular access team of nurses and interventional radiology. Triple-lumen PICC is not available for placement at our institution, but if present on admission, it is maintained. We also compared the incidence of CLABSI stratified by lumen type. This intervention went live September 29, 2022, on all adult, inpatient units to include critical care areas. CLABSI definitions from National Healthcare and Safety were applied.^6^ The monthly proportion of single-lumen PICCs placed among the total number of PICCs was displayed using a statistical process control P chart with QI Macros (Denver, CO). This study was approved by the institutional review board.

## Results

**Figure 1.**
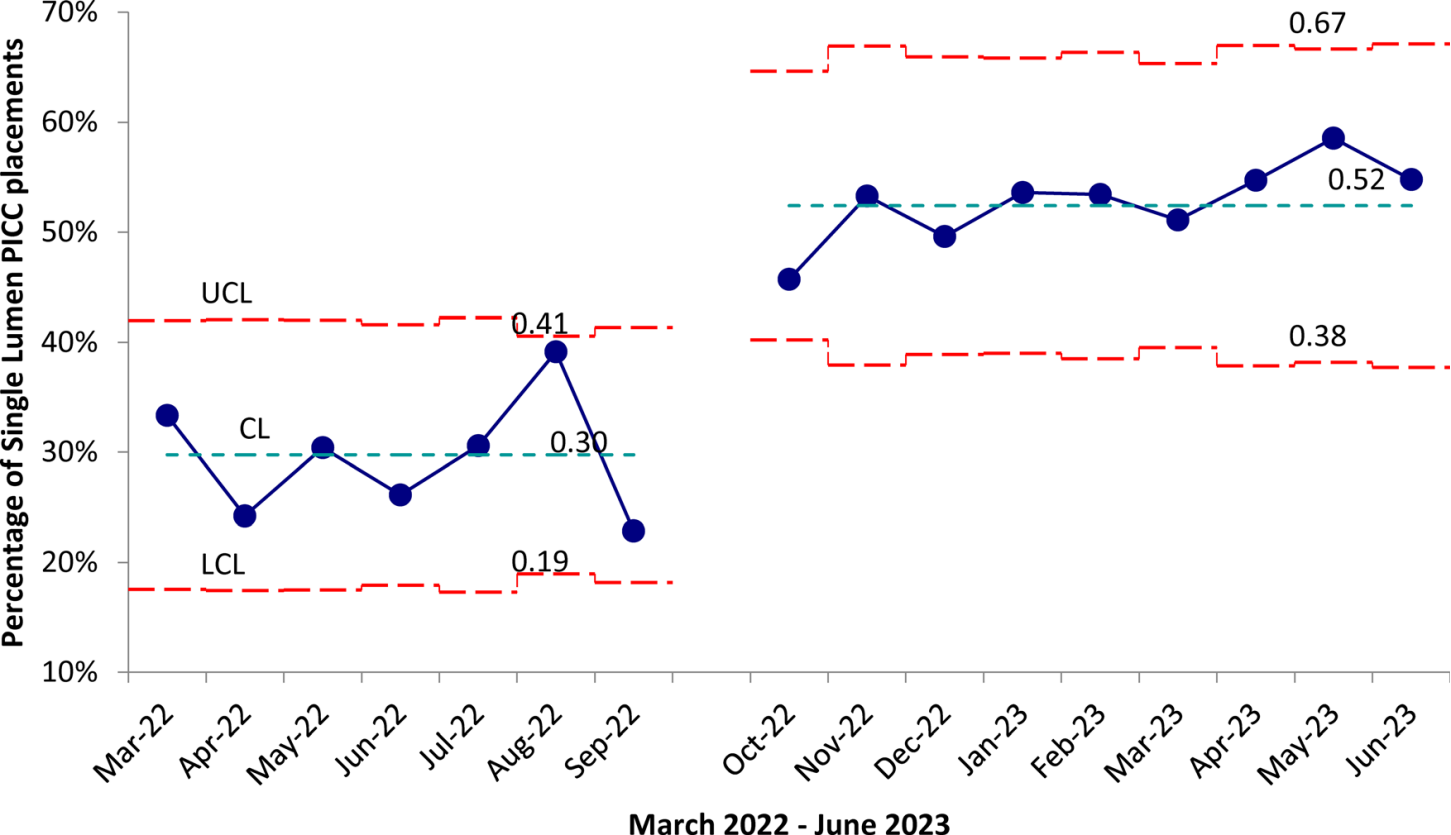
Proportion of single-lumen peripherally inserted central catheter (PICC) placements at a Midwestern academic center 2022–2023. Statistical process control p-chart was used to assess common and special cause variation using QI Macros (2023) software package. We used IHI Healthcare standards for run chart. Intervention was implemented end of September 2022. Proportions of single-lumens PICCs post-intervention, revealed nine consecutive months above the process centerline, suggesting a special cause.

Single-lumen PICC insertions increased after the EMR change implementation. The proportion of single-lumen PICCs rose from 44/126 (33%) in March 2022 to 57/104 (55%) in June 2023 (Figure 1). This increase in single-lumen PICCs post-intervention was statistically significant (*p* < 0.001, chi-square test). During the post-intervention period, the proportion of single-lumen PICCs remained relatively stable, ranging from 46% to 59%. There was no increase in non-PICC central line days (Supplemental Table 1).

**Figure 2.**
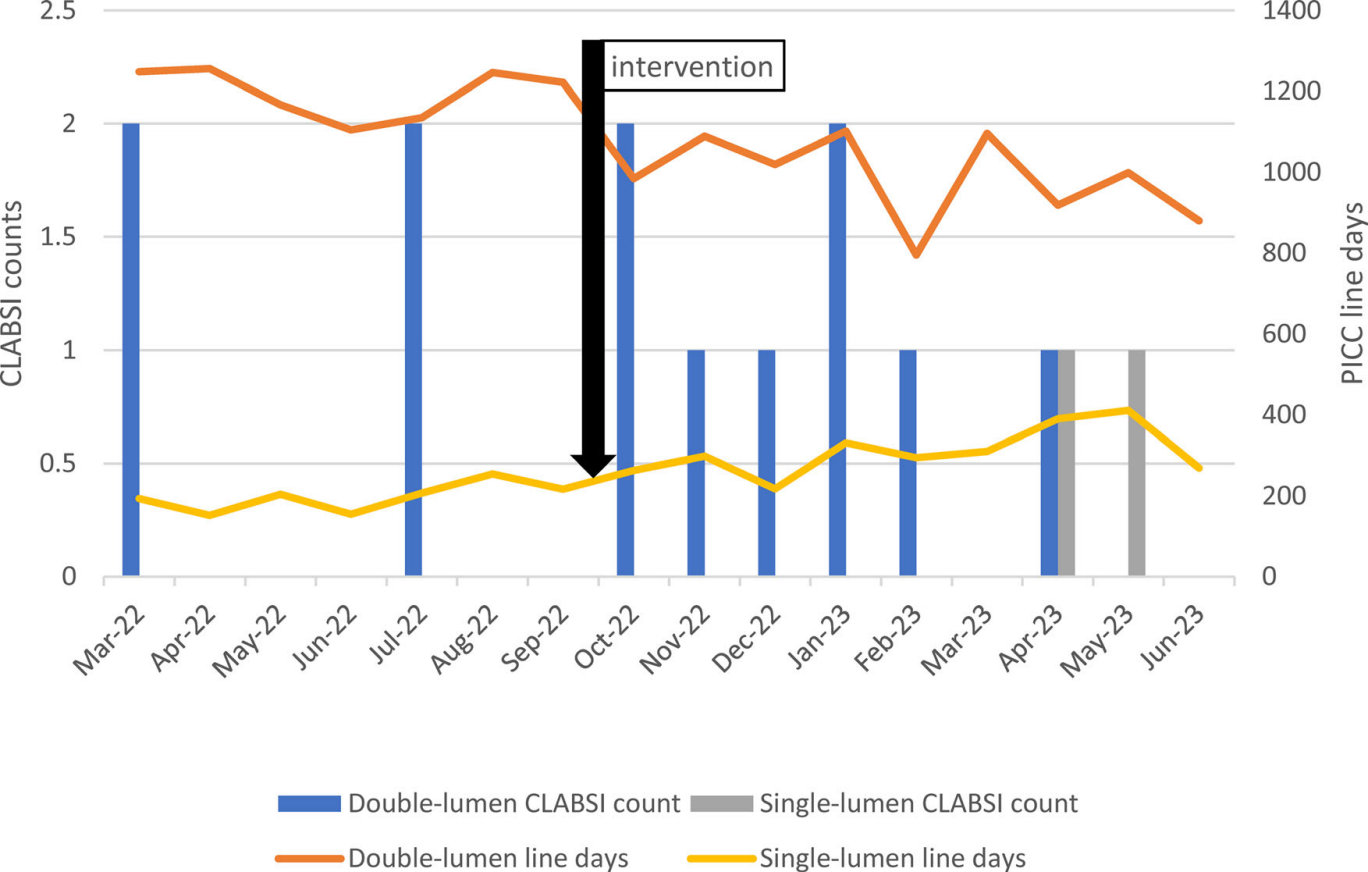
Number of central line-associated blood stream infections (CLABSI) and line days stratified by lumen number at a Midwestern academic center 2022–2023.

As a result of the new implementation, the line-days of double-lumen PICCs decreased by 29.5%, going from 1,248 days in March 2022 to 880 days in June 2023 (Figure 2). Over the study period, there were a total of 12 CLABSIs associated with double-lumen PICCs and 2 CLABSIs associated with single-lumen PICCs. The CLABSI rate was lower in single-lumen PICCs (0.48 CLABSI per 1,000 line-days) compared to double-lumen PICCs (0.70 CLABSI per 1000 line-days). However, this difference was not statistically significant at a *p*-value of 0.2 (Fisher exact test). No overall decline in CLABSI was observed after the new implementation.

## Discussion

Our intervention of defaulting to a single lumen over a double lumen in the order for PICC insertion resulted in a relative change in the placement of single-lumen PICCs increasing by 67%. Although it was not statistically significant, a lower incidence of CLABSIs was observed among patients with single-lumen PICCs compared to those with double-lumen PICCs. These findings highlight the effectiveness of improving EMR ordering strategies to promote the use of single-lumen PICCs. This helped resource utilization and potentially contributed to better patient outcomes by reducing the risk of CLABSI. Based on these results, healthcare facilities should consider implementing similar strategies to optimize PICC selection and enhance patient safety in central line management.

There have been similar studies demonstrating the effectiveness of intervention in PICC insertion order.^3,7,8^ Our absolute change of a 22% increase in single-lumen PICC use was similar to the finding in a paper recently published by Alaiev et al., who conducted the same intervention across 11 U.S. hospitals, resulting in a 24% increase.^7^ However, the study by Alaiev et al. demonstrated the post-intervention proportion of single-use PICC was around 70% while ours was ∼50%. In addition, another study published by Bozaan et al. in 2019 demonstrated the post-intervention proportion of single-lumen PICC was 94%, using a multimodal intervention, which included not only changing the electronic PICC order to a single-lumen PICC as default but also having the vascular access team evaluate the appropriateness of single vs. double lumen using Michigan Appropriateness Guide for Intravenous Catheters (MAGIC) criteria.^8,9^ Future studies should aim to also include MAGIC criteria in addition to the EMR “nudge,” given that there might still be room to even increase the use of single-lumen PICCs.

Multiple studies have established a connection between the number of lumens in a PICC and the device’s size (gauge) with subsequent complications.^2,9,10^ Moreover, current guidelines for device selection and placement advise using the minimum number of lumens necessary to fulfill clinical requirements.^9^ For example, prior simulation studies suggest that for every 1,000 PICCs inserted annually, increasing the proportion of single-lumen PICCs by 10% may prevent up to one PICC-related deep venous thrombosis and one PICC-related catheter infection per year.^11^ Our study showed a lower CLABSI rate in single-lumen PICC compared to double-lumen PICC without a statistically significant difference. We did not observe a decrease in overall CLABSI events after the intervention. The recent study conducted at 11 different U.S. hospitals by Alaiev et al. also revealed a nonsignificant decrease in CLABSI.^7^ These findings are also consistent with Baxi et al. whom showed a difference between three versus one lumen but not two as compared to one lumen.^5^ Further studies are needed to determine the true effect of single-lumen PICCs on CLABSI compared to double-lumen PICCs.

This study has several limitations. We did not perform a manual chart review to investigate other contributing factors among those with CLABSI. We also did not apply MAGIC criteria to assess the appropriateness of single- or double-lumen PICC placements, as there may have been patients who did or did not require a PICC based on clear indications or if a peripheral IV would have sufficed. Additionally, we did not assess the PICC-associated complication rate (e.g., thrombosis) between single lumen vs. double lumen. We did not observe a decrease in overall CLABSI. This analysis did not adjust for other known CLABSI risk factors that might have contributed to this stable CLABSI rate.

In conclusion, the modification to the EMR system successfully increased the utilization of single-lumen PICCs, simultaneously decreasing the use of double-lumen PICCs. Single-lumen PICCs had a nonsignificant lower number of CLABSIs per 1,000 line-days as compared to double-lumen PICCs. Healthcare facilities should not only monitor the location and types of central lines but also track the trend in the number of lumens. If the use of single-lumen PICCs is not optimized, they could consider defaulting the choice of single lumen with concomitant educational initiatives aimed at providing clear eligibility criteria for the least number of lumens needed.

## Supporting information

Trautman et al. supplementary material 1 Trautman et al. supplementary material

Trautman et al. supplementary material 2 Trautman et al. supplementary material

## Data Availability

Available upon request.

## Supplementary material

The supplementary material for this article can be found at https://doi.org/10.1017/ash.2023.511.
